# MAPPING MENTAL HEALTH PATHWAYS AMONG BISEXUAL WOMEN, MEN, AND NONBINARY INDIVIDUALS IN MID AND LATER LIFE

**DOI:** 10.1093/geroni/igad104.1552

**Published:** 2023-12-21

**Authors:** Sarah Jen, Hyun Kang, Hyun-Jun Kim, Olivia Lafountain, Karen Fredriksen-Goldsen

**Affiliations:** University of Kansas, Lawrence, Kansas, United States; George Mason University, Fairfax, Virginia, United States; University of Washington, Seattle, Washington, United States; University of Kansas, Lawrence, Kansas, United States; University of Washington, Seattle, Washington, United States

## Abstract

The health-related pathways of bisexual individuals are most often compared to those of lesbians and gay men or examine participants of different genders separately. However, little is known regarding how the health-related pathways of bisexual individuals of diverse genders compare to one another, particularly in mid- to later life. This analysis examines identity-related and social factors which impact the mental health outcomes of midlife and older bisexual women, men and non-binary individuals and compares health-related pathways across genders. Analyses examined data from the National Health, Aging, and Sexuality/Gender Study (NHAS), including bisexual women (96), men (102), and non-binary individuals (16) ages 50+. The study utilized structural equation modeling to investigate direct and indirect associations between gender and mental health via sexual identity-related (victimization, negative identity perception, and outness) and social factors (LGBTQ community engagement). For both men and women, negative identity perception was associated with being less out, particularly among women. Bisexual men who reported more victimization also reported more negative identity perception. However, negative identity perception was associated with worse mental health only among women. Across genders, victimization negatively impacted mental health and identity outness increased community engagement. Community engagement mediated the relationship between identity outness and mental health among men. Findings indicate distinct mental health pathways across genders. Thus, mental health interventions might also differ. While LGBTQ community engagement might be particularly beneficial to bisexual men, bisexual women might struggle more with identity perception. Future research will benefit from larger subsamples, particularly among non-binary individuals.

